# Anti-Allergic Properties of Curine, a Bisbenzylisoquinoline Alkaloid

**DOI:** 10.3390/molecules20034695

**Published:** 2015-03-13

**Authors:** Jaime Ribeiro-Filho, Márcia Regina Piuvezam, Patrícia T. Bozza

**Affiliations:** 1 Laboratório de Imunofarmacologia, Instituto Oswaldo Cruz, FIOCRUZ, Rio de Janeiro, RJ 21040-360, Brazil; E-Mail: jaimeribeirofilho@gmail.com; 2 Laboratório de Imunofarmacologia, Departamento de Fisiologia e Patologia, UFPB, João Pessoa, Pb 58051-900, Brazil; E-Mail: mrpiuvezam@ltf.ufpb.br

**Keywords:** curine, anti-allergic, asthma, calcium, mast cells, eosinophils

## Abstract

Curine is a bisbenzylisoquinoline alkaloid isolated from *Chondrodendron platyphyllum* (Menispermaceae). Recent findings have shed light on the actions of curine in different models of allergy and inflammation. Here we review the properties and mechanisms of action of curine focusing on its anti-allergic effects. Curine pre-treatment significantly inhibited the scratching behavior, paw edema and systemic anaphylaxis induced by either ovalbumin (OVA) in sensitized animals or compound 48/80, through mechanisms of mast cell stabilization and inhibition of mast cell activation to generate lipid mediators. In addition, oral administration of curine significantly inhibited eosinophil recruitment and activation, as well as, OVA-induced airway hyper-responsiveness in a mouse model of asthma, through inhibition of the production of IL-13 and eotaxin, and of Ca^2+^ influx. In conclusion, curine exhibit anti-allergic effects in models of lung, skin and systemic allergy in the absence of significant toxicity, and as such has the potential for anti-allergic drug development.

## 1. Curine, a Bisbenzylisoquinoline Alkaloid

The alkaloids comprise the largest class of natural products derived from plants. These substances are produced by the secondary metabolism of plants and several studies indicate that these metabolites are characterized by a great diversity of pharmacological activities. Alkaloids are alkaline substances containing one or more nitrogen atoms, usually in combination, as part of a cyclic system [1]. According to Barbosa-Filho and collaborators [2], in the twentieth century, the anti-inflammatory activities of more than 170 alkaloids has been investigated, and 140 of them were shown to be pharmacologically active in different experimental models, most of these compounds belonging to the isoquinoline alkaloids class.

The bisbenzylisoquinoline alkaloids (BBA) constitute a large group of natural products widely distributed in plants of several families, including: Menispermaceae, Berberidaceae, Lauraceae, Annonaceae, Ranunculaceae, Hernandiaceae, Magnoliaceae and Nymphaeaceae [3]. These substances are characterized by presenting two benzylisoquinoline subunits, each one with three rings, joined by one or more ether bridges [4]. Several studies have shown that BBA exhibit immunomodulatory activities in inflammatory and allergic reactions [5,6]. Recently, our research group has investigated the therapeutic potential of natural products, and promising results have been obtained in particular with regard to the use of *Cissampelos sympodialis* EICHL (Menispermaceae) and the BBA warifteine in animal models of inflammation and allergy. The immunomodulatory effects produced by the plant extract, as well as warifteine include inhibition the proliferation of splenocytes and increased IL-10 production [7], inhibition of production of ovalbumin (OVA) -specific IgE [8] and inhibition of anaphylactic shock induced by OVA [9]; inhibition of recruitment and activation of eosinophils *in vivo*, associated with reduced production of eotaxin and CysLTs [10]; inhibition of mast cell activation *in vitro* [11], in addition to modulation of AHR and of airway remodeling in experimental asthma model [12].

**Figure 1:**
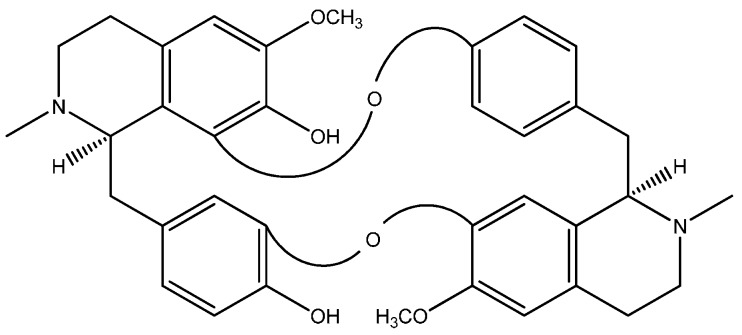
Chemical structure of curine.

Curine (Figure 1), is a BBA isolated from *Chondrodendron platyphyllum* (Menispermaceae), a plant found in northeastern Brazil, that is popularly known as “abútua”, and used in folk medicine to treat malaria, fever, pain, swelling, urethritis, cystitis and ulcers [13,14]. At least three alkaloids, including curine, isocurine, and 12-*O*-metilcurine, have been identified from this plant, and curine is the major constituent [15]. Dias *et al*. [15] have demonstrated that curine and isocurine have a vasodilator effects and have suggested that the effects of curine were associated with the inhibition of calcium channels. Medeiros *et al.* [16] demonstrated that curine decreased intracellular Ca^2+^transients in A7r5 rat thoracic aorta-derived cells. In these cells, the Ca^2+^ influx is mainly dependent on voltage-dependent Ca^2+^ channels [17] and although these cells express both l-type and T-type Ca^2+^ channels [18], in their experimental conditions, the authors demonstrated that curine effects resulted from blockade of l-type Ca^2+^ channels [16]. However, details of the blocking mechanism, as well as the selectivity of curine on different Ca^2+^ channels, including T-type Ca^2+^ channels, remain to be investigated.

Investigating the possible toxicity of curine in Swiss mice, we demonstrated that oral treatment with curine for seven consecutive days doses up to 10 times higher than the ED_50_ in mice, induced no changes in hematological parameters (such as the number of leukocytes, platelets and red blood cells, and hematocrit values and hemoglobin) or biochemical (including the concentrations of alkaline phosphatase, alanine transaminase, aspartate transaminase, bilirubin, creatinine kinase, creatinine, cholesterol, glucose, total protein and uric acid). In addition, treatment with curine did not induce the formation of gastric ulcers, and no physical or behavioral changes were observed, indicating that, in these conditions curine showed no toxicity [19].

Since curine presented interesting pharmacological properties, and its structure is very similar to the warifteine’s structure we hypothesized that this alkaloid could be an interesting target for research in anti-allergic drug development. We carried out *in vivo* and *in vitro* studies, using mouse models of allergy to determine the pharmacological properties of curine in these models. In this paper we review the roles of curine on allergy, as well as the mechanisms underlying its pharmacological effects.

## 2. An Overview of Allergy

Allergic disorders result from an exacerbated immune response to substances which are innocuous for most people. The most common allergic diseases include asthma, rhinoconjunctivitis, sinusitis, food allergy, atopic dermatitis, angioedema, urticaria, anaphylaxis and allergy to drugs and insects [20]. The etiology of these disorders is complex and is associated with a genetic susceptibility to mount IgE-mediated responses to specific environmental stimuli, a condition known as atopy [21,22,23].

The allergic reactions to specific antigens require a prior step known as sensitization, which consists in a series of events that result in the production of IgE and their binding to high-affinity Fcε receptors (FcεRI) on mast cells or basophils in the tissue [24]. In this process, dendritic cells (DCs) recognize, capture and are activated by the allergen [25,26,27]. This process induces changes in the expression of several proteins including MHC (major histocompatibility complex) class II and co-stimulatory molecules such as CD80 (B7-1) and CD86 (B7-2) that are critical for antigen presentation to Th0 lymphocytes [23]. The signaling pathway induced by the interaction between MHC class II molecule and TCR, and between co-stimulatory molecules and CD 28 expressed by lymphocytes, stimulates the translocation of the Nuclear factor of activated T-cells (NFAT1), a transcriptional factor that induces the expression of the GATA binding protein 3 (GATA 3), a major regulator in the differentiation of Th0 to Th2 lymphocytes. This process is crucially involved in the development of allergic responses because the Th2 lymphocytes secrete cytokines that orchestrate the long term inflammatory response, such as IL-13 to IL-4 and IL-5 [28].

The differentiation of Th2 cells is also facilitated by epithelium-secreted cytokines, such as (IL)-25, IL-33 and thymic stromal lymphopoietin (TLSP), which stimulate the production of IL-4 (in particular by basophils), which plays an essential role on Th2 cytokine gene activation [29]. In the context of allergic sensitization, IL-13 and IL-4 secreted by Th2 lymphocytes, induce immunoglobulin class switching to IgE production (by activated B lymphocytes). Once produced and secreted, IgE binds to FcεRI receptors on mast cells and basophils [20].

The allergic reactions are initiated by stimuli that trigger mast cell activation, causing degranulation and release of pre-formed inflammatory mediators such as histamine, and several cytokines and chemokines. These early released products, initiate the late phase which involves the recruitment of inflammatory cells such as macrophages, eosinophils, neutrophils and CD4+ T lymphocytes, especially Th2 lymphocytes [30].

Under allergic conditions the signaling of the IgE-FcεRI complex triggers a cascade of biochemical events that leads to mast cell activation and degranulation [30,31]. However, it has been demonstrated that mast cells can be activated by synthetic compounds such as calcium channel agonists and compound 48/80, which are important experimental tools in allergy [32]. Mast cell activation involves elevation of cytosolic Ca^2+^ concentration, followed by activation of kinases such as protein kinase C (PKC), mitogen-activated protein kinase (MAPK) and phosphatidylinositol 3-kinase (PI3K) pathway [33], with subsequent translocation of vesicles containing the granules and, ultimately, fusion with the plasma membrane, mediated anchoring by proteins of the family SNAREs [34,35].

Allergic reactions involving the sudden systemic release of mediators, may trigger a clinical condition known as anaphylactic shock [36]. Anaphylactic shock is a severe reaction in which the person experiences symptoms such as shortness of breath, hypotension, cardiac arrhythmia, vomiting, urticaria, headache and unconsciousness, it may result in death [37].

The pathophysiological features of allergic diseases are directly influenced by the environment in which they occur. Thus, a key feature of most skin allergies is itching [38]. In this context, the itching is mainly resulting from stimulation of specific receptors in neurons for a large variety of mediators such as histamine, serotonin, substance P, LTB_4_, IL-31, prostaglandins and proteases [39,40]. Severe itching is a major problem because it induces the scratching behavior resulting in the formation of skin lesions that worsen the situation of allergic individuals [41].

**Figure 2:**
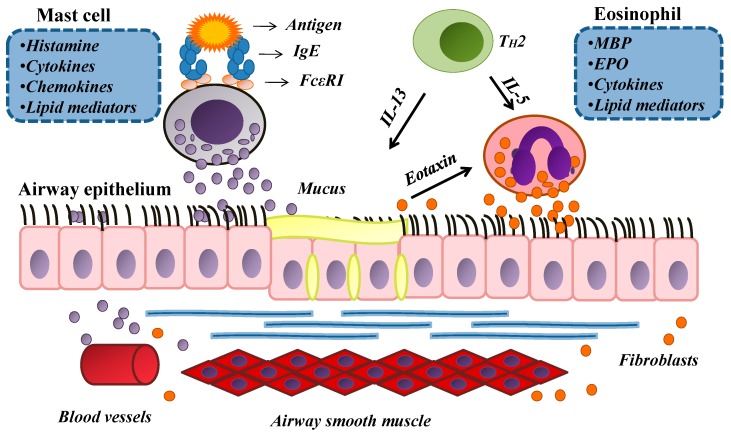
Pathophysiological Mechanisms in Asthma.

Allergic asthma (Figure 2) is a disease characterized by chronic airway inflammation that is associated with an intense recruitment and activation of leukocytes, tissue remodeling and airway hyperreactivity (AHR) [29,42]. AHR is a key feature of asthma episodes. This condition is defined as a narrowing of the airways in response to nonspecific stimuli, which usually do not affect the airways of normal subjects [42,43]. The AHR is the result of complex interactions between activated leukocytes and smooth muscle of airline routes and previous studies have shown that inflammatory mediators, including IL-13 and cysteinyl leukotrienes (CysLTs) play important roles in this process [44,45].

In regard to the recruitment and activation of leukocytes, noticeable accumulation of eosinophils is a characteristic feature of allergic inflammation where it play roles in the pathogenesis of allergic diseases due to their capacity to release an array of immunomodulatory and tissue-damaging mediators [46,47,48].

These cells are recruited to sites of allergic inflammation and activated in response to stimulation mostly by IL-5 and eotaxin. Once activated, eosinophils release toxic proteins (major basic protein (MBP), eosinophil cationic protein (ECP), eosinophil peroxidase (EPO), and eosinophil-derived neurotoxin (EDN)), as well as an array of cytokines (including IL-13 and IL-4) through well-regulated and differential secretion from their granules [47]. Eosinophils are also a major source of newly formed lipid mediators that amplify the inflammatory response. Vieira, de-Abreu *et al*. [49] demonstrated that eotaxin effectively induces both recruitment and activation of eosinophils, increasing the generation of lipid droplets in these cells. Although these organelles can be found in non-stimulated eosinophils, an increased formation of lipid droplets during allergic inflammation is important for the production of eicosanoids, specially CysLTs that cause various inflammatory effects including: bronchoconstriction, mucus hypersecretion, increased microvascular permeability, bronchial hyperresponsiveness and eosinophil infiltration [50,51]. Thus, the recruitment and subsequent activation of eosinophils at sites of allergic inflammation are key events in the pathogenesis of asthma and other allergic diseases.

Allergic diseases usually require long-term therapy, and many drugs are currently available for allergy management. Histamine receptor antagonists and mast cell stabilizers are currently used to treat many allergic symptoms, including edema and itch. However, they are not effective in the management of numerous allergic conditions, including in allergic asthma [52,53]. The drugs that are widely used in the treatment of asthma today include inhaled corticosteroids (anti-inflammatory drugs that regulate the expression of cytokines, chemokines, lipid mediators and adhesion molecules), long-acting β_2_-adrenergic agonists, usually used in combination with corticosteroids and leukotriene modifiers (drugs that prevent the effects of leukotrienes in asthma pathogenesis) [54]. Phosphodiesterase inhibitors, cytokine-targeted monoclonal antibodies, and allergen-specific immunotherapies are also important in asthma therapy and other allergic diseases [20,55]. Despite the variety of anti-allergic therapies currently available, the demand for new treatments for the development of safe and effective drugs for the treatment of asthma and other allergic diseases remains a major research field.

## 3. The Anti-Allergic Effects of Curine

### 3.1. The Effects of Curine on Experimental Allergic Asthma

**Figure 3:**
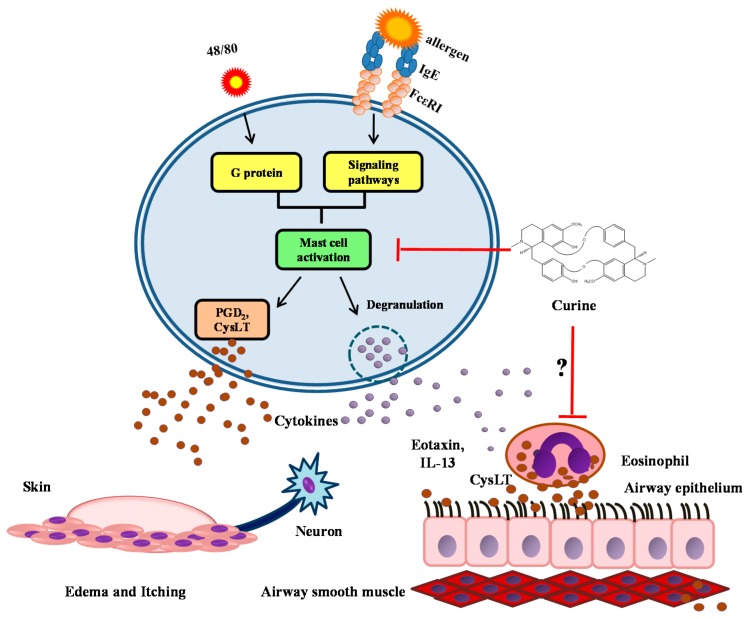
Mechanisms of action of curine in allergy.

Our research team was the first to demonstrate the anti-allergic properties of curine. We used a well-established mouse model of asthma, in which Balb/c mice were sensitized intraperitoneally (i.p.) with ovalbumin + Al(OH)_3_ on days 1 and 10, and from day 19 to day 24 after sensitization, mice were challenged daily for 20 min with OVA (5%) in PBS by aerosol [52]. Eosinophilic infiltration into the airways is a hallmark of this model, since allergic challenge induces eosinophil maturation and differentiation from precursors in the bone marrow and their migration to sites of inflammation in response to mediators such as eotaxin and IL-5 [46]. Treating the animals with different oral doses of curine, we observed that such treatment dose-dependently inhibited the number of eosinophils in the BAL, with a median effective dose (ED_50_) of 0.8 mg/Kg demonstrating the inhibitory role that curine plays on eosinophil recruitment. Additionally, the eosinophils from the curine-treated group presented decreased number of cytoplasmic lipid bodies, compared to the OVA-challenged and non-treated group. It has been shown that increased lipid body assembly within eosinophils is closely associated with cellular activation and LTC_4_ production in an allergic response and therefore plays important roles in inflammatory conditions and may be used as a marker of eosinophil activation [47,49]. Therefore, the reduced number of eosinophils in the BAL, associated with the reduced number of lipid bodies in these cells, indicated that curine plays an inhibitory role on both migration and activation of eosinophils (Figure 3). Of note, curine leads to decreased production of eotaxin in allergic-challenged animals, which was associated with the reduced eosinophil recruitment and lipid body formation in eosinophils, suggesting that curine inhibition of eotaxin production as a mechanism involved in the observed reduction in eosinophil recruitment and activation [49]. The analysis of AHR, a particular feature of asthma [43], revealed that curine decreased the observed Penh values to levels similar to those observed in the animals treated with dexamethasone. Moreover, curine also inhibited the production of IL-13, a key mediator of AHR and other allergic responses. Indeed, accumulating evidence have demonstrated that IL-13, is significantly involved in development of AHR [44,56,57], and in the modulation of various pathophysiological aspects of asthma, such as class switching to IgE, mucus production, inflammation, airway remodeling and the contraction of airway smooth muscle (ASM) through an interaction with specific receptors on the cell surface [58].

Medeiros and colleagues [16] demonstrated that curine might have a direct effect on l-type Ca^2+^channels in vascular smooth muscle cells and, in light of allergic mechanisms, calcium-dependent signaling pathways are required for activation of many cell types, including leukocytes and epithelial and airway muscle cells [29], with significant impact on eosinophilic inflammation and AHR. Accordingly, recent work has demonstrated that verapamil, a calcium channel antagonist, presented significant inhibitory effects on a model of allergic asthma [59]. Thus, the involvement of calcium-dependent mechanisms on the anti-allergic effects of curine was investigated. We demonstrated that curine pre-treatment significantly inhibited a calcium-induced trachea contractile response *ex vivo*, suggesting that curine inhibits the influx of calcium by blocking voltage-dependent Ca^2+^channels in rat tracheal smooth muscle. Interestingly, in non-allergic mice, curine did not alter the bronchoconstriction induced by methacholine (*in vivo*), which is mediated by calcium release from intracellular stores [60]. These data supported the hypothesis that curine inhibits calcium channels in the cell membrane. Additionally, *in vivo* treatments with curine or verapamil (a l-type calcium-channel antagonist) using the same dose, time and method of administration had similar effects on AHR and eosinophilic inflammation, suggesting that the anti-allergic effects of curine may be, at least in part, dependent on the inhibition of calcium influx.Finally, curine post-treatment was effective in the inhibition of eosinophil recruitment and activation, highlighting the therapeutic potential of curine as an anti-allergic compound.

### 3.2. The Effects of Curine on Mast Cell-Dependent Responses

Following the results obtained in the mouse model of allergic asthma, we investigated the effectiveness of curine in inhibiting mast cell-dependent responses in mice [61]. Allergic reactions are associated with the activation and release of mast cell products [62], including pre-formed mediators, such as histamine and serotonin; and the synthesis of cytokines and lipid mediators, including prostaglandin D_2_ (PGD_2_) and CysLTs through specific signaling pathways [30,63]. Using a model of mast cell activation induced by compound 48/80 *in vivo*, we demonstrated that curine pre-treatment significantly inhibited the scratching behavior. Itching is a key feature of most skin allergies [38], which can result in the formation of skin lesions [41]. In allergic reactions, itch is mostly dependent on histamine release by mast cells, although these cells can also release other mediators, such as adenosine triphosphate (ATP), tryptase, CysLTs, leukotriene B_4_ (LTB), IL-31 and prostaglandins, that significantly activate nerve fibers [64,65]. Therefore, the modulation of scratching behavior by curine suggests that this alkaloid inhibits mast cell-dependent responses in mice.

Edema is another important feature of allergic inflammation [66]. Using models of mouse paw edema induced by compound 48/80 or histamine administration, it was demonstrated that curine had an anti-allergic effect similar to cromoglycate (a mast cell stabilizer drug), but different from promethazine (H_1_ receptor antagonist), suggesting that curine's effects may result from the inhibition of mast cell degranulation, instead of the inhibition of histamine at allergic sites. In animals sensitized and challenged with OVA, curine and verapamil significantly inhibit edema and that there were no differences between these treatments. Additionally, in acute allergic pleurisy, induced by sensitization and challenge with OVA, curine and verapamil decreased the concentrations of PGD_2_ and CysLT in the pleural washes of OVA-stimulated mice. These data highly corroborate the effects of curine in the model of asthma, and because previous studies have shown that mast cells are a major source of CysLTs and PGD_2_ in the acute phase of allergy [67], it was suggested that curine and verapamil inhibited the production of these mediators, at least in part, through a direct effect on mast cell activation (Figure 3). This was confirmed by experiments using rat basophilic leukemia (RBL) -2H3 cells stimulated *in vitro* with IgE anti-dinitrophenol (DNP). In this cells curine and verapamil partially inhibited mast cell degranulation, while exhibited expressive inhibition of PGD_2_ and CysLT production, indicating that curine inhibits mast cell activation by modulating both degranulation and newly synthesized lipid mediator production.

Finally, a single oral pre-treatment with curine delayed the death by anaphylactic shock in mice stimulated with either OVA or compound 48/80; and when OVA was given in a dose on which the untreated group presented mortality <100%), curine reduced the total mortality, and similar results were obtained with verapamil administered under the same conditions, suggesting that both curine and verapamil modulate the anaphylactic shock reaction in mice. Together, these results demonstrated that curine inhibits mast cell-dependent responses by modulating mast cell activation through mechanisms that may involve the inhibition of a calcium dependent response.

## 4. Other Pharmacological Effects of Curine

The anti-allergic activity, specially, regarding to leukocyte recruitment and activation, and the ethnopharmacological data, which indicate that *C. platyphyllum* is a plant popularly used to treat inflammatory symptoms, suggested that curine could present anti-inflammatory and analgesic effects. Using different protocols in mice [68], we demonstrated that oral pre-treatment with curine significantly inhibited mouse paw edema induced by carrageenan and zymosan, and acetic acid-induced vascular permeability, suggesting that curine inhibits edema formation by modulating the inflammatory reaction. Pre-treatment with curine also inhibited both the writhing response induced by acetic acid and the licking behavior in the inflammatory phase of the formalin test. Nevertheless, curine did not inhibit the neurogenic phase of the formalin test, indicating that curine has an analgesic effect that is associated with anti-inflammatory mechanisms, rather than being associated with a direct effect on neuronal activation. Accordingly, in these experiments, curine presented phenotypic outcomes similar to those observed for indomethacin, a non-steroidal antiinflammatory drug (NSAID), but different from morphine, a central acting analgesic drug. In addition, curine significantly inhibited the hyperalgesic response triggered by carrageenan (which is highly dependent on the action of PGE_2_ [69,70] and inhibited PGE_2_ production without affecting COX-2 expression, indicating that the analgesic and anti-inflammatory effects of curine result, at least in part, from the inhibition of PGE_2_ production. In fact, classical NSAIDs and many natural products promote the inhibition of COX activity without alteration in the expression of this enzyme [71,72]. Accordingly, because of the modulation of calcium influx, curine may affect many inflammatory signaling pathways, including those involved in the production of PGE_2_ metabolism, particularly PLA_2_ [72,73]. However, the molecular mechanisms involved in the anti- inflammatory and analgesic effects of curine remain to be better elucidated.

## 5. Concluding Remarks

Accumulating findings have demonstrated that orally administered curine exhibited significant anti-allergic effects in different animal models in the absence of detected toxicity, which suggest this alkaloid as a promising candidate for further investigation and development. The effectiveness of the post-treatment with curine on eosinophil recruitment and activation, suggest that this alkaloid might have pro-resolution properties, which are now under investigation. The association between the anti-allergic effects of curine with its action of calcium influx modulation, may open new perspectives in the search for new candidates to anti-allergic drugs. In conclusion, curine present anti-allergic properties that are, at least in part associated with inhibition of calcium-dependent responses, and, as such, has the potential for the development of anti-allergic drugs.

## References

[1] Harborne J.B. (1984). Phytochemical Methods: A Guide to Modern Techniques of Plant Analysis.

[2] Barbosa-Filho J.M., Piuvezam M.R., Moura M.D., Silva M.S., Batista-Lima K.V., Leitão-da-Cunha E.V., Fechine I.M., Takemura O.S. (2006). Anti-inflammatory activity of alkaloids: A twenty-century review. Braz. J. Pharmacogn..

[3] Tolkachev O.N., Nakova E.P., Evstigneeva R.P. (1977). Bisbenzylisoquinoline alkaloids. Chem. Nat. Prod..

[4] Barbosa-Filho J.M. (1997). Quimiodiversidade e Potencialidade Farmacológica da Flora Paraibana. Cad. Farm..

[5] Seow W.K., Li S.T.Y., Thong Y.H. (1986). Inhibitory effect of tetrandrine on human neutrophil and monocyte adherence. Immunol. Lett..

[6] Teh B.S., Seow W.K., Li S.Y., Thong Y.H. (1990). Inhibition of prostaglandin and leukotriene generation by the plant alkaloids tetrandrine and berbamine. Int. J. Immunopharmacol..

[7] Piuvezam M.R., Peçanha L.M., Alexander J., Thomas G. (1999). *Cissampelos sympodialis* Eichl. leaf extract increases the production of IL-10 by concanavalin-A treated BALB/c spleen cells. J. Ethnopharmacol..

[8] Bezerra-Santos C.R., Balestieri F.M., Rossi-Bergmann B., Peçanha L.M., Piuvezam M.R. (2004). *Cissampelos sympodialis* Eichl. (Menispermaceae): Oral treatment decreases IgE levels and induces a Th1-skewed cytokine production in ovalbumin-sensitized mice. J. Ethnopharmacol..

[9] Bezerra-Santos C.R., Peçanha L.M.T., Piuvezam M.R. (2005). *Cissampelos sympodialis* Eichl. (Menispermaceae) inhibits anaphylactic shock reaction in murine allergic model. Braz. J. Pharmacog..

[10] Bezerra-Santos C.R., Vieira-de-Abreu A., Barbosa-Filho J.M., Bandeira-Melo C., Piuvezam M.R., Bozza P.T. (2006). Anti-allergic properties of *Cissampelos sympodialis* and its isolated alkaloid warifteine. Int. Immunopharmacol..

[11] Costa H.F., Bezerra-Santos C.R., Barbosa-Filho J.M., Martins M.A., Piuvezam M.R. (2008). Warifteine, a bisbenzylisoquinoline alkaloid, decreases immediate allergic and thermal hyperalgesic reactions in sensitized animals. Int. Immunopharmacol..

[12] Bezerra-Santos C.R., Vieira-de-Abreu A., Vieira G.C., Ribeiro-Filho J., Barbosa-Filho J.M., Pires A.L., Martins M.A., Souza H.S., Bandeira-Melo C., Bozza P.T. (2012). Effectiveness of *Cissampelos sympodialis* and its isolated alkaloid warifteine in airway hyperreactivity and lung remodeling in a mouse model of asthma. Int. Immunopharmacol..

[13] Correa P.M. (1984). Dicionário das plantas úteis do Brasil e das exóticas cultivadas.

[14] Gotfredsen E. Liber Herbarum II: Chondrodendron platyphyllum (A.St.Hil.) Miers. http://www.liberherbarum.com.

[15] Dias C.S., Barbosa-Filho J.M., Lemos V.S., Cortes S.F. (2002). Mechanisms involved in the vasodilator effect of curine in rat resistance arteries. Planta Med..

[16] Medeiros M.A., Pinho J.F., de-Lira D.P., Barbosa-Filho J.M., Araújo D.A., Cortes S.F., Lemos V.S., Cruz J.S. (2011). Curine, a bisbenzylisoquinoline alkaloid, blocks l-type Ca^2+^ channels and decreases intracellular Ca^2+^transients in A7r5 cells. Eur. J. Pharmacol..

[17] Medeiros M.A., Nunes X.P., Barbosa-Filho J.M., Lemos V.S., Pinho J.F., Roman-Campos D., de Medeiros I.A., Araújo D.A., Cruz J.S. (2009). (S)-reticuline induces vasorelaxation through the blockade of l-type Ca^2+^ channels. Naunyn Schmiedebergs Arch. Pharmacol..

[18] Hall J., Jones R.D., Jones T.H., Channer K.S., Peers C. (2006). Selective inhibition of l-type Ca^2+^ channels in A7r5 cells by physiological levels of testosterone. Endocrinology.

[19] Ribeiro-Filho J., Calheiros A.S., Vieira-de-Abreu A., Carvalho K.I.M., Mendes D.S., Bandeira-Melo C., Martins M.A., Dias C.S., Piuvezam M.R., Bozza P.T. (2013). Curineinibits eosinophil activation and airway hyper-responsiveness in a mouse model of allergic asthma. Toxicol. Appl. Pharmacol..

[20] Holgate S.T., Polosa R. (2008). Treatment strategies for allergy and asthma. Nat. Rev. Immunol..

[21] Allen D.B. (2006). Effects of inhaled steroids on growth, bone metabolism, and adrenal function. Adv. Pediatr..

[22] Sicherer S.H., Sampson H.A. (2007). Peanut allergy: Emerging concepts and approaches for an apparent epidemic. J. Allergy Clin. Immunol..

[23] Murdoch J.R., Lloyd C.R. (2010). Chronic inflammation and asthma. Mutat. Res..

[24] Willart M.A.M., Hammad H. (2010). Alarming Dendritic Cells for Allergic Sensitization. Allergol. Int..

[25] Lambrecht B.N., Salomon B., Klatzmann D., Pauwels R.A. (1998). Dendritic cells are required for the development of chronic eosinophilic airway inflammation in response to inhaled antigen in sensitized mice. J. Immunol..

[26] Chomarat P., Dantin C., Bennett L., Banchereau J., Palucka A.K. (2003). TNF skews monocyte differentiation from macrophages to dendritic cells. J. Immunol..

[27] Yadav R., Zammit D.J., Lefrancois L., Vella A.T. (2006). Effects of LPS-mediated bystander activation in the innate immune system. J. Leukoc. Biol..

[28] Rodriguez-Palmero M., Hara T., Thumbs A., Hunig T. (1999). Triggering of T cell proliferation through CD28 induces Gata-3 and promotes T helper type 2 differentiation *in vitro* and *in vivo*. Eur. J. Immunol..

[29] Paul W., Zhu J. (2010). How are TH2-type immune responses initiated and amplified. Nat. Rev. Immunol..

[30] Gould H.J., Sutton B.J. (2008). IgE in allergy and asthma today. Nat. Rev. Immunol..

[31] Galli S.J., Maurer M., Lantz C.S. (1999). Mast cells as sentinels of innate immunity. Curr. Opin. Immunol..

[32] Tatemoto K., Nozaki Y., Tsuda R., Konno S., Tomura K., Furuno M., Ogasawara H., Edamura K., Takagi H., Iwamura H. (2006). Immunoglobulin E independent activation of mast cell is mediated by Mrg receptors. Biochem. Biophys. Res. Commun..

[33] Huber M., Hughes M.R., Krystal G. (2000). Thapsigargin-induced degranulation of mast cells is dependent on transient activation of phosphatidylinositol-3 kinase. J. Immunol..

[34] Puri N., Kruhlak M.J., Whiteheart S.W., Roche P.A. (2003). Mast cell degranulation requires Nethylmaleimide-sensitive factor-mediated SNARE disassembly. J. Immunol..

[35] Gilfillan A.M., Tkaczyk C. (2006). Integrated signalling pathways for mast-cell activation. Nat. Rev. Immunol..

[36] Kemp S.F., Lockey R.F. (2002). Anaphylaxis: A review of causes and mechanisms. J. Allergy Clin. Immunol..

[37] Wade J.P., Liang M.H., Sheffer A.L. (1989). Exercise-induced anaphylaxis: Epidemiologic observations. Prog. Clin. Biol. Res..

[38] Buddenkotte J., Steinhoff M. (2010). Pathophysiology and therapy of pruritus in allergic and atopicdiseases. Allergy.

[39] Garibyan L., Rheingold C.G., Lerner E.A. (2013). Understanding the pathophysiology of itch. Dermatol. Ther..

[40] Greaves M.W. (2010). Pathogenesis and Treatment of Pruritus. Curr. Allergy Asthma Rep..

[41] Wahlgren C.F. (1991). Itch and atopic dermatitis: Clinical and experimental studies. Acta Derm. Venereol..

[42] Barnes P.J. (2008). Immunology of asthma and chronic obstructive pulmonary disease. Nat. Rev. Immunol..

[43] Lauzon A.-M., Bates J.H.T., Donovan G., Tawhai M., Sneid J., Sanderson M.J. (2012). A multi-scale approach to airway hyperresponsiveness: From molecule to organ. Front. Physiol..

[44] Wills-Karp M., Luyimbazi J., Xu X., Schofield B., Neben T.Y., Karp C.L., Donaldson D.D. (1998). Interleukin-13: Central mediator of allergic asthma. Science.

[45] Capra V., Thompson M.D., Sala A., Cole D.E., Folco G., Rovati G.E. (2007). Cysteinyl leukotrienes and their receptors in asthma and other inflammatory diseases: Critical update and emerging trends. Med. Res. Rev..

[46] Gleich G.J., Adolphson C.R., Leiferman K.M. (1993). The biology of the eosinophilic leukocyte. Annu. Rev. Med..

[47] Akuthota P., Xenakis J.J., Weller P.F. (2011). Eosinophils: Offenders or General Bystanders in Allergic Airway Disease and Pulmonary Immunity?. J. Innate Immun..

[48] Rothenberg M.E., Hogan S.P. (2006). The eosinophil. Annu. Rev. Immunol..

[49] Vieira-de-Abreu A., Assis E.F., Gomes G.S., Castro-Faria-Neto H.C., Weller P.F., Bozza P.T., Bandeira-Melo C. (2005). Allergic Challenge-Elicited Lipid Bodies Compartmentalize *in Vivo* Leukotriene C4 Synthesis within Eosinophils. Am. J. Respir. Cell Mol. Biol..

[50] Lewis R.A., Austen K.F., Soberman R.J. (1990). Leukotrienes and other products of the 5-lipoxygenase pathway. Biochemistry and relation to pathobiology in human diseases. N. Engl. J. Med..

[51] Laitinen L.A., Laitinen A., Haahtela T., Vilkka V., Spur B.W., Lee T.H. (1993). Leukotriene E4and granulocytic infiltration into asthmatic airways. Lancet.

[52] Cook E.B., Stahl J.L., Barney N.P., Graziano F.M. (2002). Mechanisms of antihistamines and mast cell stabilizers in ocular allergic inflammation. Curr. Drug Targets Inflamm. Allergy.

[53] Kim K. (2012). Neuroimmunological Mechanism of Pruritus in Atopic Dermatitis Focused on the Role of Serotonin. Biomol. Ther..

[54] Szefler S.J. (2011). Advancing asthma care: The glass is only half full. J. Allergy Clin. Immunol..

[55] Edwards A.M., Howell J.B. (2000). The chromones: History, chemistry and clinical development. A tribute to the work of Dr REC. Altounyan. Clin. Exp. Allergy.

[56] Lloyd C.M., Gonzalo J.A., Nguyen T., Delaney T., Tian J., Oettgen H., Gutierrez-Ramos J.C., Coyle A.J. (2001). Resolution of bronchial hyperresponsiveness and pulmonary inflammation is associated with IL-3 and tissue leukocyte apoptosis. J. Immunol..

[57] Kuperman D.A., Lewis C.C., Woodruff P.G., Rodriguez M.W., Yang Y.H., Dolganov G.M., Fahy J.V., Erle D.J. (2005). Dissecting asthma using focused transgenic modeling and functional genomics. J. Allergy Clin. Immunol..

[58] Bloemen K., Verstraelen S., van-Den-Heuvel S., Witters H., Nelissen I., Schoeters G. (2007). The allergic cascade: Review of the most important molecules in the asthmatic lung. Immunol. Lett..

[59] Khakzad M.R., Mirsadraee M., Mohammadpour A., Ghafarzadegan K., Hadi R., Saghari M., Meshkat M. (2012). Effect of verapamil on bronchial goblet cells of asthma: An experimental study on sensitized animals. Pulm. Pharmacol. Ther..

[60] Foster R.W., Okpalugo B.I., Small R.C. (1984). Antagonism of Ca^2+^ and other actions of verapamil in guinea-pig isolated trachealis. Br. J. Pharmacol..

[61] Ribeiro-Filho J., Leite F.C., Costa H.F., Calheiros A.S., Torres R.C., de Azevedo C.T., Martins M.A., Dias C.S., Bozza P.T., Piuvezam M.R. (2014). Curine inhibits mast cell-dependent responses in mice. J. Ethnopharmacol..

[62] Liew F.Y., Pitman N.I., McInnes I.B. (2010). Disease-associated functions of IL-33: The new kid in the IL-1 family. Nat. Rev. Immunol..

[63] Cockcroft D.W., Hargreave F.E., O’byrne P.M., Boulet L.P. (2007). Understanding allergic asthma from allergen inhalation tests. Canad. Resp. J..

[64] Hossen M.A., Inoue T., Shinmei Y., Minami K., Fujii Y., Kamei C. (2006). Caffeic Acid Inhibits Compound 48/80-Induced Allergic Symptoms in Mice. Biol. Pharm. Bull..

[65] Nakanishi M., Furuno T. (2008). Molecular Basis of Neuroimmune Interaction in an *in Vitro* Coculture Approach. Cell. Mol. Immunol..

[66] Ash A.S., Schild H.O. (1997). Receptors mediating some actions of histamine. Br. J. Pharmacol..

[67] Boyce J.A. (2003). The role of mast cells in asthma. Prostaglandins Leukot. Essent. Fatty Acids.

[68] Leite F.C., Ribeiro-Filho J., Costa H.F., Salgado P.R., Calheiros A.S., Carneiro A.B., de Almeida R.N., Dias Cda S., Bozza P.T., Piuvezam M.R. (2014). Curine, an Alkaloid Isolated from *Chondrodendron platyphyllum* Inhibits Prostaglandin E_2_ in Experimental Models of Inflammation and Pain. Planta Med..

[69] Tjølsen A., Berge O.G., Hunskaar S., Rosland J.H., Hole K. (1992). The formalin test: an evaluation of the method. Pain.

[70] Hunskaar S., Hole K. (1987). The formalin test in mice: Dissociation between inflammatory and non-inflammatory pain. Pain.

[71] Vane J.R., Botting R.M. (1996). Mechanism of action of anti-inflammatory drugs. Scand. J. Rheumatol..

[72] Van Rossum D.B., Patterson R.L. (2009). PKC and PLA_2_: Probing the complexities of the calcium network. Cell Calcium.

[73] Rainsford K.D. (2007). Anti-inflammatory drugs in the 21st century. Subcell. Biochem..

